# How Do We Evaluate Health in All Policies? Comment on "Developing a Framework for a Program Theory-Based Approach to Evaluating Policy Processes and Outcomes: Health in All Policies in South Australia"

**DOI:** 10.15171/ijhpm.2018.31

**Published:** 2018-04-08

**Authors:** Ditte Heering Holt, Nanna Ahlmark

**Affiliations:** National Institute of Public Health, University of Southern Denmark, Copenhagen, Denmark.

**Keywords:** Health in All Policies, Theory-Based Evaluation, Intersectoral Policymaking, Policy Process

## Abstract

It is well-established that population health is influenced by a multitude of factors, many of which lie outside the scope of the health sector. In the public health literature it is often assumed that intersectoral engagement with nonhealth sectors will be instrumental in addressing these social determinants of health. Due to the expected desirable outcomes in population health, several countries have introduced Health in All Policies (HiAP). However, whether this systematic, top-down approach to whole-of-government action (which HiAP entails) is efficient in changing government policies remains unclear. A systematic evaluation of HiAP is therefore much needed. Lawless and colleagues present an evaluation framework for HiAP in their article: "Developing a Framework for a Program Theory-Based Approach to Evaluating Policy Processes and Outcomes: Health in All Policies in South Australia." This work is an important endeavor in addressing this problem (of uncertainty as to whether HiAP is effective) and represents an essential contribution to the HiAP literature. Nonetheless, in the spirit of encouraging ongoing reflection on this topic, we wish to highlight some challenges in the presented framework, which may pose difficulties in operationalization. We find that the evaluation framework faces two main limitations: its unclear causal logic and its level of complexity. We argue that in order to function as a tool for evaluation, the framework should be explicit about the mechanisms of change and enable us to trace whether the assumed causal relations resulted in changes in practice. Developing manageable evaluation frameworks, albeit simplified, may then be an important part of cumulating the theoretical insights aspired in theory-based evaluation. On this basis, we highlight how HiAP processes and healthy public policies respectively involve different mechanisms, and thus argue that different program theories are needed.

In a recent article, Lawless and colleagues^1^ present an evaluation framework for Health in All Policies (HiAP) in South Australia. This is a timely and much needed contribution to the literature on HiAP, where no systematic evaluation has yet been published. Although HiAP is often advocated as a necessary approach in order to address the social determinants of health,^2,3^ it remains an open question to what extent (if at all) a top-down approach to whole-of-government action, such as HiAP, is efficient in changing government policies, and whether this contributes to achieving better population health and health equity outcomes.

To a great extent, the lack of published evaluations on this issue may be attributed to the high complexity of policy processes and consequently the difficulties in establishing an adequate and manageable evaluation framework. While this relates to methodological questions regarding how to design evaluations, it also involves underlying ontological questions regarding how HiAP may be conceptualized.

For instance, de Leeuw et al^4^ present HiAP as a reincarnation of healthy public policies and thus conceptualize HiAP as health policy. Based on this understanding, they argue that policy – and thus HiAP – should not be analyzed as an intervention. They find that the study of HiAP would benefit from using political science theory rather than intervention research to capture the ‘messiness’ of policy processes. The question for evaluation based on policy analysis may be: How can we understand policy change?

A different conceptualization of HiAP is presented by Carey et al,^5^ who define HiAP as an “instrumental process-based intervention” (IPI). This conceptualization is rooted in public administration research and focuses attention on the institutional arrangements, like the Health Lens Analysis and the Cabinet Taskforce, which attempt to increase coordination and intersectoral engagement to impact on (healthy) public policies. Conceptualizing HiAP as an IPI refocuses attention from the overall policy process to the new governance structures and decision-making processes introduced by the top-down approach to whole-of-government action. This conceptualization makes theory-based evaluation a relevant approach for studying the theoretical mechanisms assumed to enable (better) intersectoral engagement (and thereby affect policy-making). The question for evaluation then becomes how HiAP (understood as new governance structures and decision-making processes) works and under which conditions it may contribute to achieving policy change for better population health and health equity.

Lawless and colleagues^1^ apply this latter conceptualization of HiAP to their study of HiAP in South Australia, which makes theory-based evaluation particularly pertinent.

Developing a program theory for HiAP (as an IPI) is, however, a challenging undertaking: policy-making processes are both “messy,” as pointed out by de Leeuw et al,^4^ and “opaque,” as highlighted by Exworthy,^6^ in the sense that they rarely take place at a single moment in time making it difficult for researchers to evaluate where, when, and how they take place. Baum et al^7^ note how evaluation of HiAP is challenging since it operates in complex dynamic systems that involve a range of sectors and disciplines, and occurs in changing political and operational contexts. Here, Baum et al draw on Rogers’^8^ distinction between complicated and complex aspects of program evaluation of interventions. Rogers propose that complicated aspects of an intervention refer to its multiple components; multiple agencies, multiple simultaneous causal links, and multiple alternative causal strands. These aspects pose logistical challenges. Complex aspects refer to the recursive causalities and emergent outcomes of interventions – that is, outcomes and the means to achieve them emerge during implementation. These aspects pose challenges in the sense that causal relations cannot be predicted.

Lawless and colleagues^1^ present a welcomed attempt to develop an evaluation framework amidst these apparent challenges. The framework succeeds in visualizing the complexity of HiAP. We find, however, that operationalization of the framework (in order to advance our understanding of the HiAP mechanisms) is difficult for two reasons: the unclear causal assumptions and the level of complexity of the framework itself.

Firstly, the causal logic is not very clear. Theory-based evaluations seek, as the authors correctly state, to make the causal assumptions behind a program explicit through a “predictive chain-of-logic.” In the present framework, arrows are drawn in a chain between the different boxes, but the logic behind the arrows, ie, the theory of change, is not fully explicated. Hence, it is neither very clear exactly how HiAP mechanisms are thought to contribute to the expected results, nor how one is to evaluate these processes. The authors note that only the strongest links are indicated by the arrows of the framework. We find, however, that the framework entails so many mutually influential layers that the causal logic is not immediately obvious. Moreover, the evaluation criteria that would help determine whether the expected results take place are missing. Consequently, we fail to see how the framework opens up the black box as described^1^ (p. 8). We find that the theoretical insights (from political and social science theory) used to build the framework according to the article are difficult to recognize in the framework. In order to use the framework as a program theory for evaluation such scientific theories could be used to both operationalize components and the relationships between them.

As presented above, HiAP also entails complex aspects. In Rogers’^8^ understanding, multiple government units use relevant opportunities as they emerge and negotiate relevant outcomes throughout the process.^8^ Hence, the causal relations may not all be defined beforehand. We believe that the framework could accommodate this complexity by being more explicit about how certain outcomes cannot be predicted while specific theoretical assumptions still guide the framework. Although the authors state that they update and evaluate their framework in an ongoing process, without explicit theoretical assumptions and evaluation criteria this will not cumulate theoretical insights but rather be an ongoing documentation of empirical developments.

Moreover, while we believe that application of theory from social and political sciences is highly relevant and important, the mixing of numerous theories into one general framework raises the question of whether and how the theories are mutually compatible in terms of their underlying philosophies of science. Clarity regarding the theoretical assumptions is therefore necessary.

The limitations of vague causal logic, mechanisms of change, and theoretical underpinning in the framework reflect a common set of challenges for theory-based evaluation though. As Weiss^9^ point out, program theories often specify activities and outcomes without specifying the theories of change.

Secondly, the framework includes such a large number of components that actual evaluation is rendered difficult, especially in light of the vague explication of the causal assumptions discussed above. With Rogers’^8^ definition in mind, HiAP is complicated in the sense that it is implemented in different sites, by different governmental units and sectors, and includes multiple components. The problem, as Rogers^8^ points out, is that “complicated interventions that have many components pose challenges to evaluation, given the limited number of variables that can be identified and empirically investigated” (p. 31). Winter^10^ also brings attention to how an (evaluation) model, if it is not specific enough but becomes too general, may in fact be an obstacle for further development of our understanding of the process, as the generality inhibits precise specification of variables and causal mechanisms. We fear that the many components hinder empirical testing as well as our understanding of the causal mechanisms.

In other words, we believe a program theory is helpful when (1) it shows a program’s causal assumptions *and* (2) provides a concrete tool that enables us to trace whether the causal assumptions in fact resulted in the assumed changes.^11^ As such, the evaluation framework by Lawless and colleagues suffers from the same inherent challenge as HiAP itself, namely that it reproduces the high level of complexity, rather than presenting a simplified tool to evaluate those aspects of HiAP that may be traced. Building on Winther,^10^ we suggest that it might be more fruitful to develop partial program theories rather than a general model. This would enable better conceptual clarification and more clarity on evaluation criteria.^10^

Hence, we suggest that the evaluation framework might gain clarity by, on the one hand, presenting only those causal assumptions which are expected to leave marks behind that may be traced, and, on the other hand, explicitly acknowledging those outcomes that are emergent and hence expected to continuously evolve.^8^ In both cases the theoretical underpinnings should be unfolded in the text and the framework in order to allow for the theoretical cumulation that feeds further theoretical development.^12^

Therefore, we suggest that the operationalization of the framework take the definition of HiAP as an IPI as the key premise. This involves that the framework may not include all assumed causal relations of the policy process, but would focus on the institutional arrangements introduced.

Moreover, the definition of HiAP as an IPI signifies that the changes to the government processes, which are sought in a HiAP approach, are not proposed to be inherently able to improve health.^5^ Rather, the output of successful HiAP would be (improved) coordination and intersectoral engagement. The outcome of successful HiAP would thus be policy change for better health, or in other words, the creation and implementation of healthy public policies. This in turn is expected to lead to better population health and health equity.

Therefore, if one wishes to evaluate whether HiAP contributes to better population health and health equity several program theories are needed. That is, at least one program theory should “explain how and why [HiAP] is thought to work”^1^ by making the theoretical propositions regarding HiAP mechanisms explicit, as discussed above. Taking the complicated aspects of HiAP into consideration, this may entail developing several partial program theories, as suggested by Winter.^10^ A second, but quite different question for evaluation relates to the impacts of healthy public policies on population health outcomes. That is, whether the resulting policy changes effect better population health. A theory-based evaluation of this question must be concerned with the various mechanisms that are thought to make the policy effective, counting diverse factors like economic structures, environmental exposure, and health practices, depending on the specific policy. However, policy evaluations involving such ‘health mechanisms’ comprise entirely different chains of logic than the mechanisms of a systematic, top-down approach to whole-of-government action, such as HiAP. Here, the HiAP-processes may rather be defined as an important contextual factor or as a question of implementation.^10^

In terms of the present framework by Lawless and colleagues,^1^ we find that the main contribution lies in its potential to further our theoretical understanding of the mechanisms introduced with HiAP. As such, the main purpose of a theory-based evaluation of HiAP should not be empirical generalization; that is, to answer the empirical question about whether HiAP improves population health and health equity. Rather, we wish to highlight the potential contribution of theory-based evaluation in terms of its ability to increase our theoretical understanding of the mechanisms introduced with HiAP and how they interact with complex policy systems in different (national) contexts in order to bring about policies for better health.

In summary, we find that the framework entails some weaknesses that render its use as an effective evaluation tool difficult. We suggest that the program theory would be made more manageable by selecting fewer causal relations and focus on those that may in fact be traced as part of the evaluation. While this simplifies reality, it may also hold the key to undertake the endeavor of evaluating at all. Moreover, developing manageable evaluation frameworks, albeit simplified, is an important part of the cumulation of theoretical insights aspired in theory-based evaluation.^12^

The framework is indeed helpful in visualizing the complex and multiple layers entailed in HiAP-processes. If one is to operationalize it as an evaluation tool, we find that a few steps in a different direction may be needed; towards simplification and explication of the theoretical mechanisms one wishes to trace.

## Ethical issues

Not applicable.

## Competing interests

Authors declare that they have no competing interests.

## Authors’ contributions

Both authors have contributed to conception and design, drafting the argument and read and agreed on the final manuscript.
